# Supplementary material for: Genome-Wide Characterization and Expression Profiling of ABA Biosynthesis Genes in a Desert Moss Syntrichia caninervis
Source: Plants (Basel). 2023 Mar 1;12(5):1114. doi: 10.3390/plants12051114 (PMC10004953; doi:10.3390/plants12051114)
Supplement: Supplementary file 1 [file plants-12-01114-s001.zip › plants-2154888-supplementary1/Supplementary/Supplementary.pdf]

Supplementary Information for

Genome-Wide Characterization and Expression Profiling of ABA biosynthesis genes in a desert moss *Syntrichia caninervis*

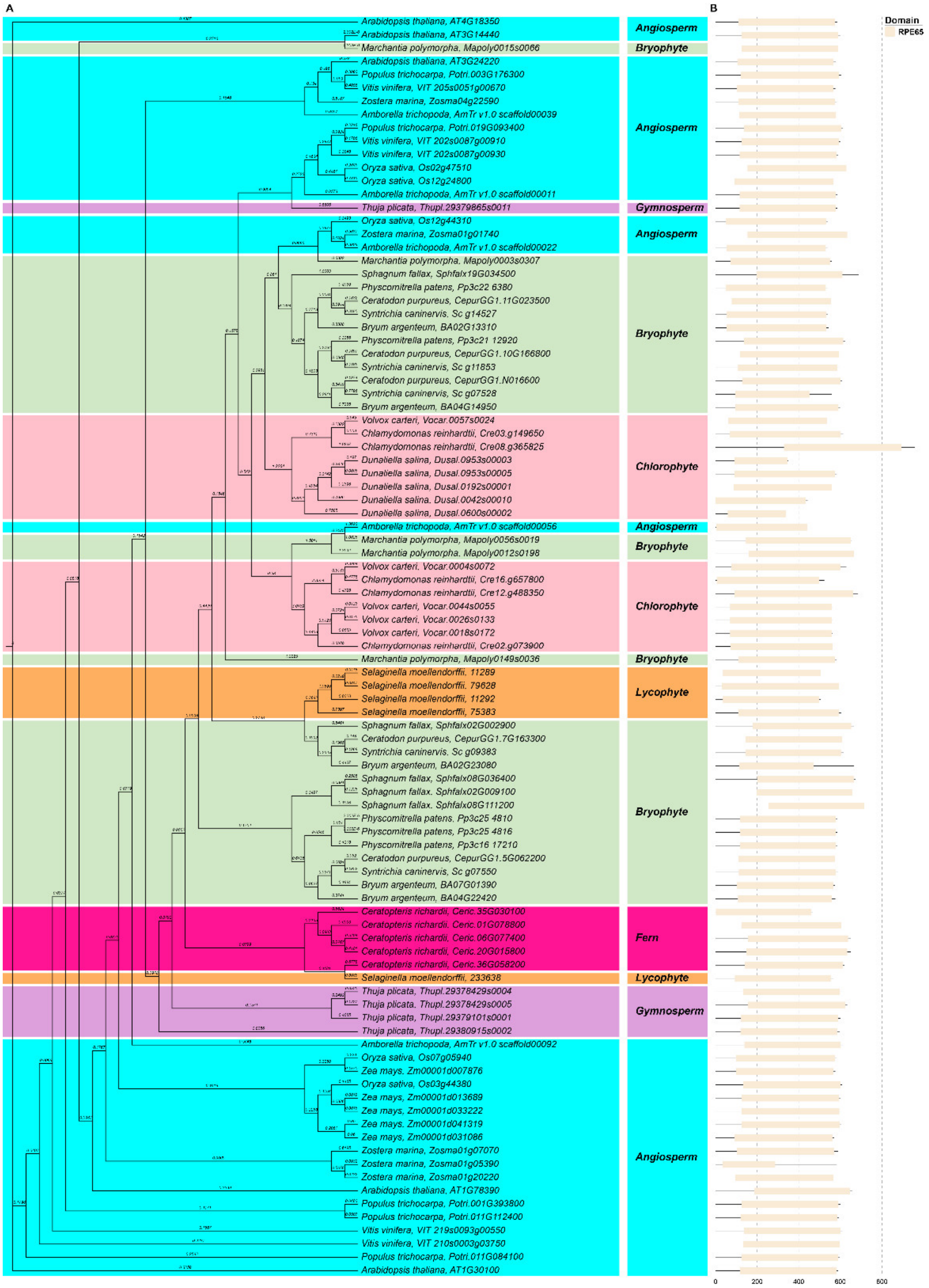

Figure S1. Phylogenetic and deduced protein structure analysis of NCED. With *Arabidopsis* NCED (AT3G14440) as a query, the NCED homologous sequences were retrieved from Phytozome using BLASTP. (A) The phylogenetic analysis of NCED; the maximum likelihood (ML) phylogenetic tree was

constructed with the full-length amino acid sequence of the *NCED* genes from 19 species in IQTree v1.6.12 using the best-fit substitution model to automatically selected by the software; background colors represent different plant taxa. (B) The conserved domain analysis of NCED; conserved motifs were identified by PFAM website, the motifs were displayed in different colors boxes.

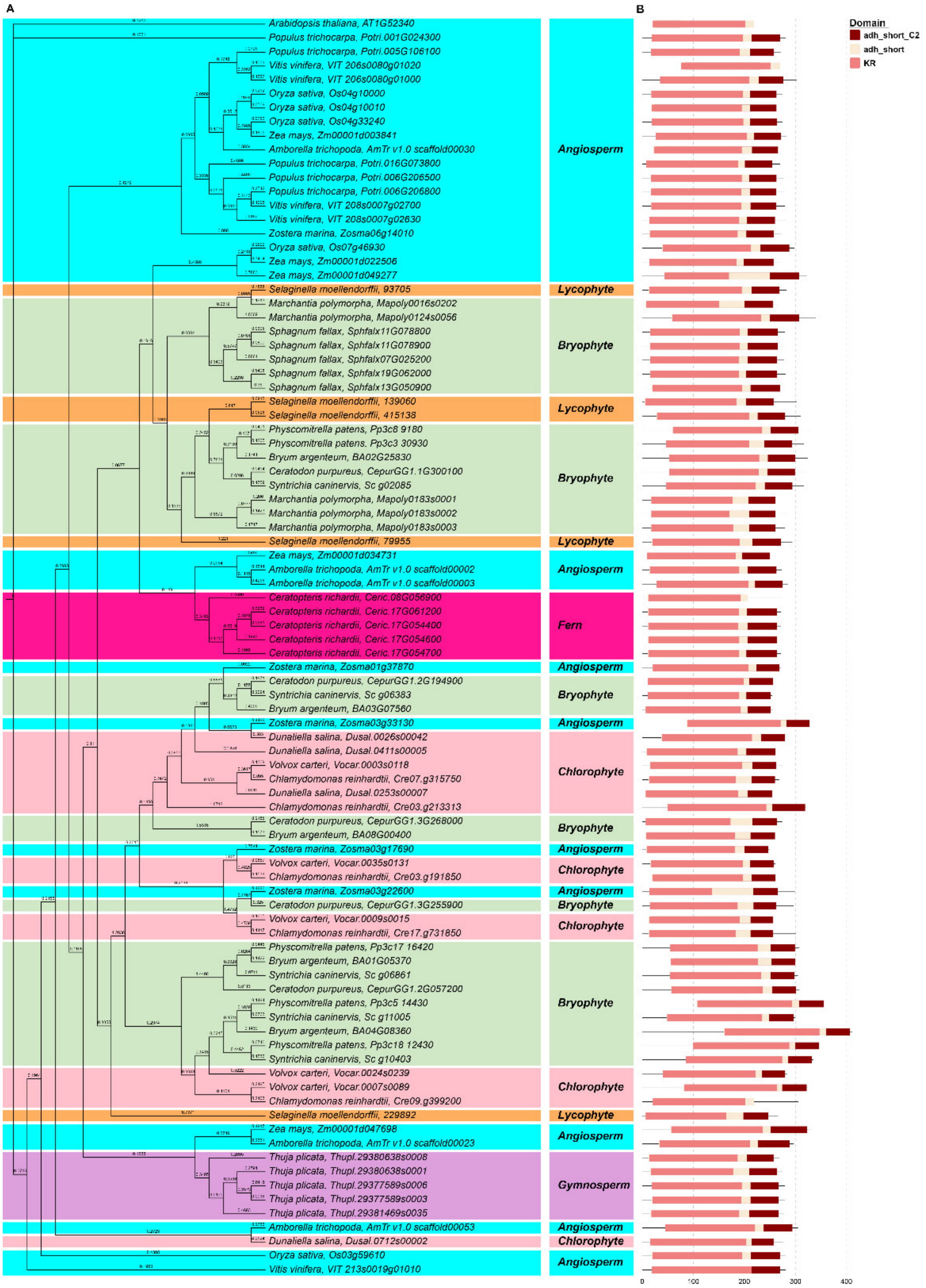

Figure S2. Phylogenetic and deduced protein structure analysis of ABA2. With *Arabidopsis* ABA2 (AT1G52340) as a query, the ABA2 homologous sequences were retrieved from Phytozome using BLASTP. (A) The phylogenetic analysis of ABA2; the phylogenetic tree was constructed with the full-length amino acid sequence of the *ABA2* genes from 19 species in IQTree v1.6.12 using the best-fit substitution model to automatically selected by the software; background colors represent different plant taxa. (B) The conserved domain analysis of ABA2; conserved motifs were identified by PFAM website, the motifs were

displayed in different colors boxes.

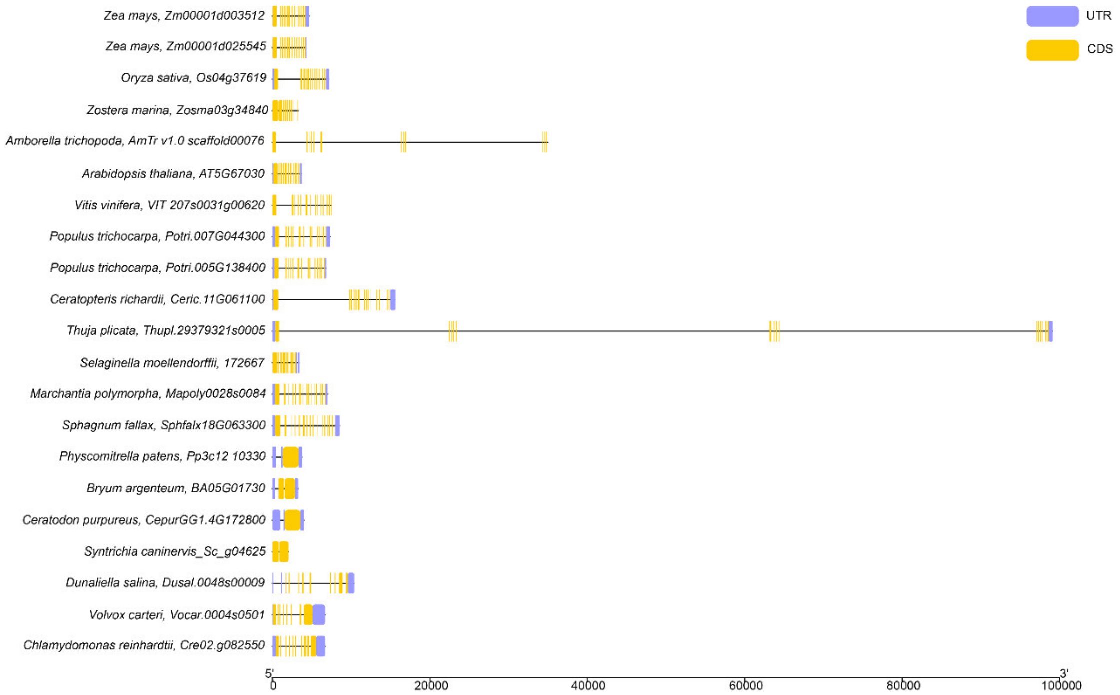

Figure S3. The intron/ exon structure of *ABA1* genes. TBtools analyzed the exon-intron structure of ABA biosynthesis genes from 19 species with default parameters. The yellow box represents the exon, the gray line represents the intron, and the purple box represents UTR.

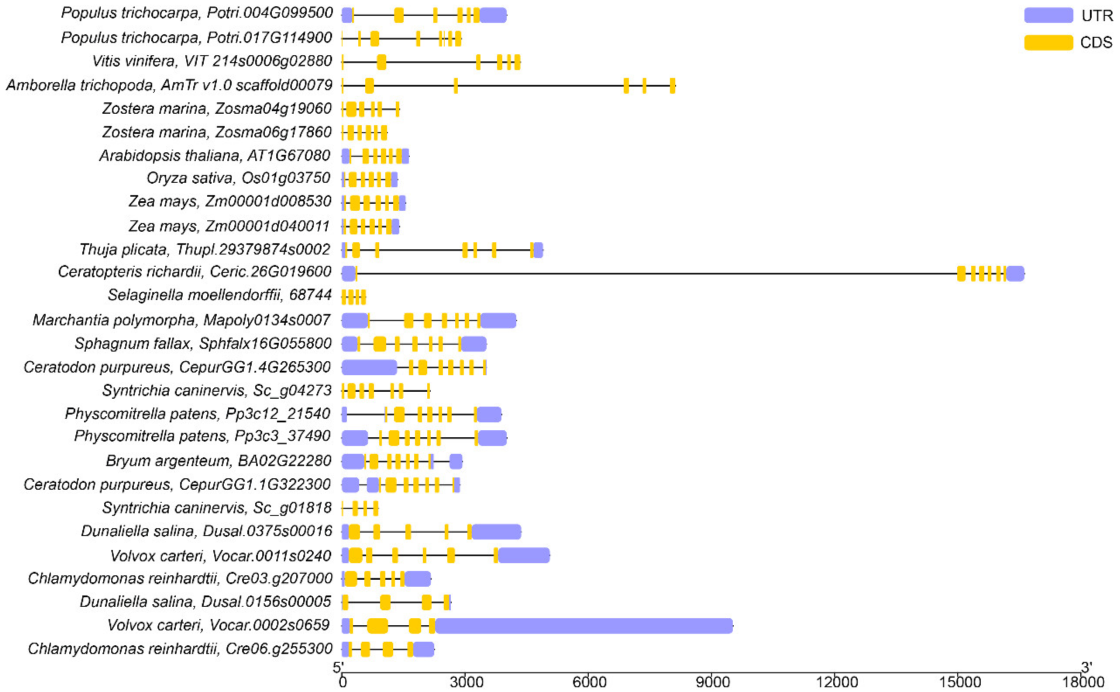

Figure S4. The intron/ exon structure of *ABA4* genes. TBtools analyzed the exon-intron structure of ABA biosynthesis genes from 19 species with default parameters. The yellow box represents the exon, the gray line represents the intron, and the purple box represents UTR.

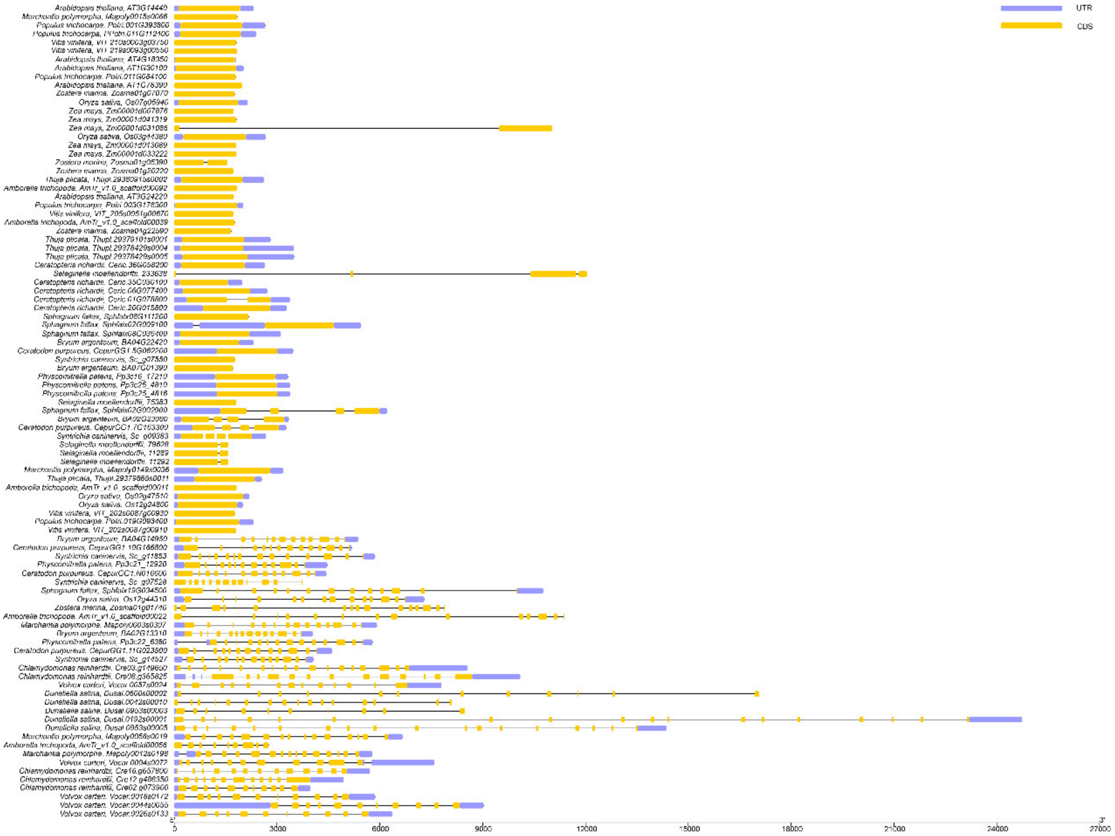

Figure S5. The intron/ exon structure of *NCED* genes. TBtools analyzed the exon-intron structure of ABA biosynthesis genes from 19 species with default parameters. The yellow box represents the exon, the gray line represents the intron, and the purple box represents UTR.

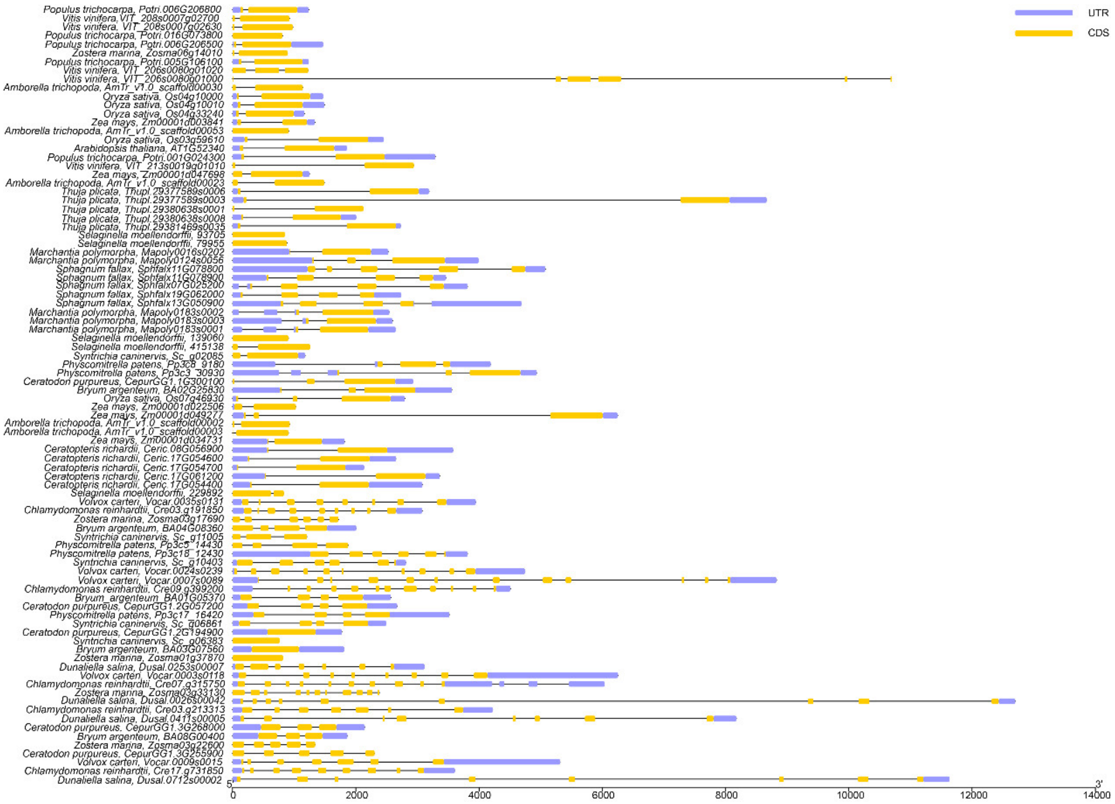

Figure S6. The intron/ exon structure of *ABA2* genes. TBtools analyzed the exon-intron structure of ABA

biosynthesis genes from 19 species with default parameters. The yellow box represents the exon, the gray line represents the intron, and the purple box represents UTR.

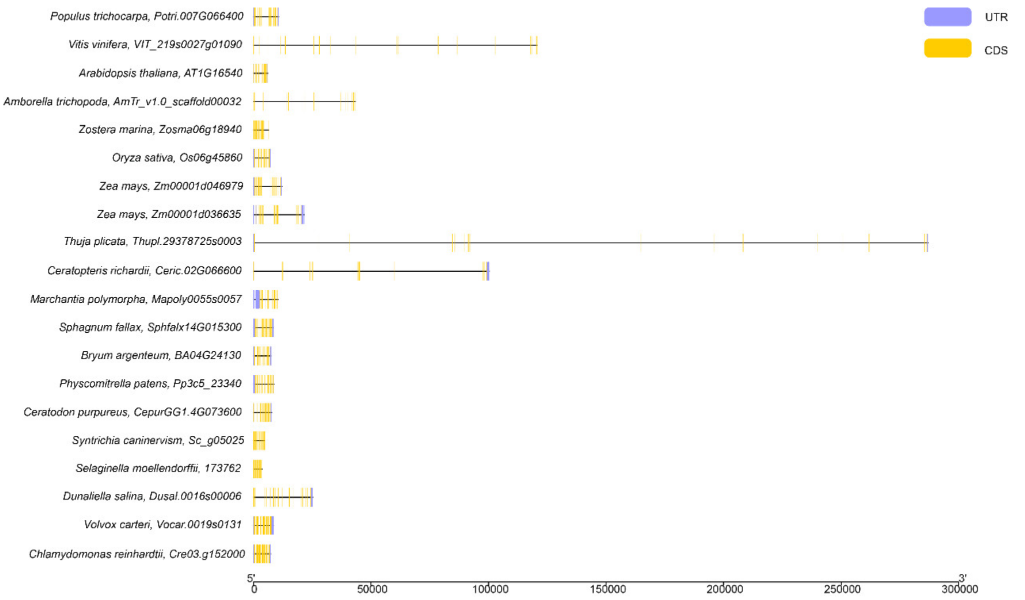

Figure S7. The intron/ exon structure of *ABA3* genes. TBtools analyzed the exon-intron structure of ABA biosynthesis genes from 19 species with default parameters. The yellow box represents the exon, the gray line represents the intron, and the purple box represents UTR.

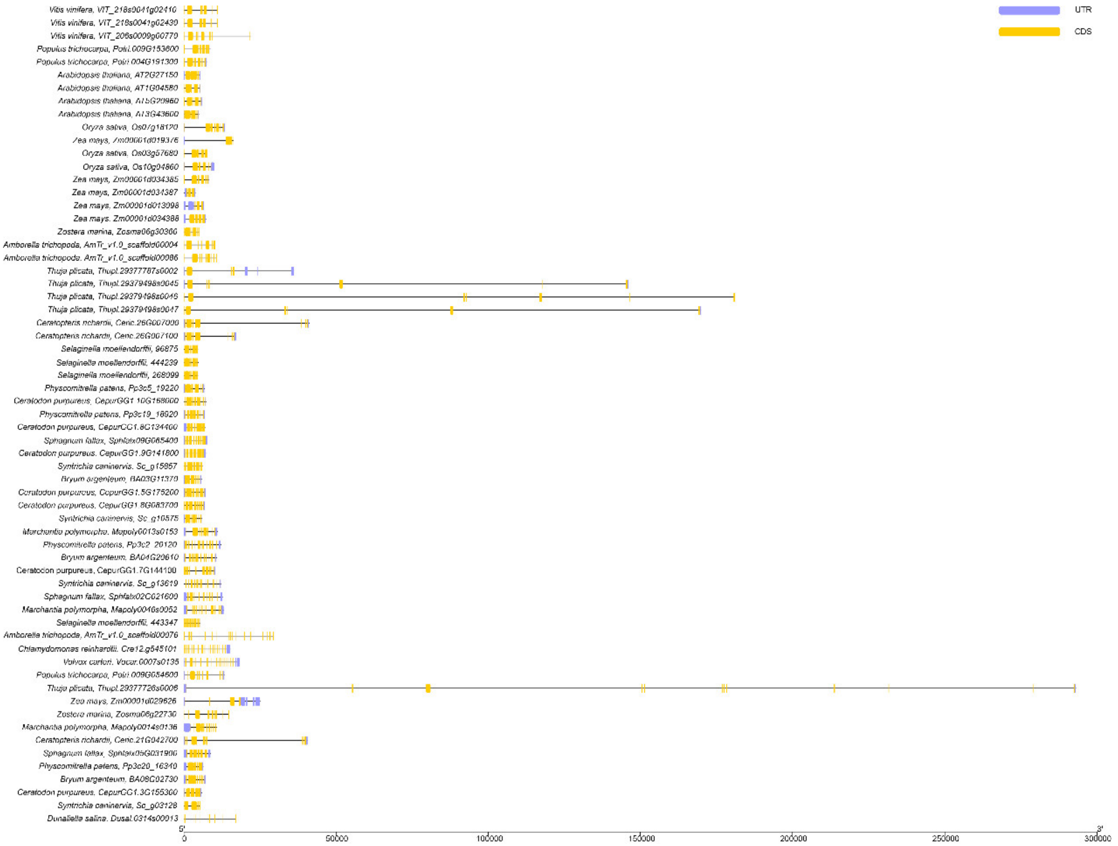

Figure S8. The intron/ exon structure of *AAO* genes. TBtools analyzed the exon-intron structure of ABA biosynthesis genes from 19 species with default parameters. The yellow box represents the exon, the gray line represents the intron, and the purple box represents UTR.
